# Supplementary material for: Surveillance of tuberculosis and treatment outcomes following screening and therapy interventions among marriage-migrants and labor-migrants from high TB endemic countries in Taiwan
Source: PeerJ. 2021 Mar 16;9:e10332. doi: 10.7717/peerj.10332 (PMC7977376; doi:10.7717/peerj.10332)
Supplement: Supplemental Information 1 [file peerj-09-10332-s001.docx]

Metadata:

1. The raw data used in this study were acquired from the TCDC TB registry system which could not be carried outside due to individual information privacy protection policy. For interested readers can go to apply for access to the data resources which is provided with related details of individual TB cases including “area, age, and gender”.

- The statistical table of determined cases on different areas, ages and gender. Available from website: <https://data.cdc.gov.tw/en/dataset/aagstable-tuberculosis>
- National tuberculosis (except multi-drug resistant tuberculosis) nationality and non-nationality statistics.

Available from website: https://nidss.cdc.gov.tw/en/nndss/disease?id=010

1. Metadata: Summary of TB notification and treatment outcomes among labor and marriage migrants.

|  | | |  | | | Labor migrants | | | | | | | | | | | |  | Marriage migrants | | | | | | |
| --- | --- | --- | --- | --- | --- | --- | --- | --- | --- | --- | --- | --- | --- | --- | --- | --- | --- | --- | --- | --- | --- | --- | --- | --- | --- |
| Year | | |  | | | 2012 | 2013 | | 2014 | | 2015 | | Sum | | | | 2012 | | | 2013 | | 2014 | 2015 | Sum | |
|  | | | Notification TB cases | | | 472 | 581 | | 646 | | 624 | | 2323 | | | | 109 | | | 92 | | 95 | 84 | 380 | |
| Age | | | <=24 | | | 101 | 102 | | 129 | | 120 | | 452 | | | | 6 | | | 9 | | 4 | 4 | 23 | |
|  | | | 25-44 | | | 363 | 464 | | 489 | | 483 | | 1799 | | | | 91 | | | 72 | | 80 | 72 | 315 | |
|  | | | <=45 | | | 8 | 15 | | 28 | | 21 | | 72 | | | | 12 | | | 11 | | 11 | 8 | 42 | |
| Sex | | | Female | | | 295 | 352 | | 361 | | 370 | | 1378 | | | | 107 | | | 92 | | 89 | 82 | 370 | |
|  | | | Male | | | 177 | 229 | | 285 | | 254 | | 945 | | | | 2 | | | 0 | | 6 | 2 | 10 | |
| Country | | | Indonesia | | | 221 | 286 | | 322 | | 296 | | 1125 | | | | 13 | | | 6 | | 7 | 9 | 35 | |
|  | | | Vietnam | | | 77 | 98 | | 102 | | 123 | | 400 | | | | 44 | | | 44 | | 34 | 30 | 152 | |
|  | | | Philippines | | | 109 | 128 | | 152 | | 162 | | 551 | | | | 4 | | | 4 | | 8 | 0 | 16 | |
|  | | | Thailand | | | 65 | 69 | | 68 | | 43 | | 245 | | | | 0 | | | 2 | | 3 | 1 | 6 | |
|  | | | China | | |  |  | |  | |  | | 0 | | | | 48 | | | 36 | | 42 | 44 | 170 | |
| Population | | Indonesia | | | | 191127 | 213234 | | 229491 | | 236526 | | 870378 | | | | 27684 | | | 27943 | | 28287 | 28699 | 112613 | |
|  | | Vietnam | | | | 100050 | 125162 | | 150632 | | 169981 | | 545825 | | | | 87357 | | | 89042 | | 91004 | 93441 | 360844 | |
|  | | Philippines | | | | 86786 | 89024 | | 111533 | | 123058 | | 410401 | | | | 7465 | | | 7707 | | 8021 | 8326 | 31519 | |
|  | | Thailand | | | | 67611 | 61709 | | 59933 | | 58372 | | 247625 | | | | 8336 | | | 8375 | | 8467 | 8525 | 33703 | |
|  | | China | | | |  |  | |  | |  | | 0 | | | | 306,514 | | | 315905 | | 323,358 | 330069 | 1275846 | |
| TB incidence | | Indonesia | | | | 115.63 | 134.13 | | 140.31 | | 125.14 | |  | | | | 46.96 | | | 21.47 | | 24.75 | 31.36 |  |  |
|  |  | Vietnam | | | | 76.96 | 78.3 | | 67.71 | | 72.36 | |  | | | | 50.37 | | | 49.41 | | 37.36 | 32.11 |  |  |
|  | | Philippines | | | | 125.6 | 143.78 | | 136.28 | | 131.65 | |  | | | | 53.58 | | | 51.9 | | 99.74 | 0 |  |  |
|  | | Thailand | | | | 96.14 | 111.82 | | 113.46 | | 73.67 | |  | | | | 0 | | | 23.88 | | 35.43 | 11.73 |  |  |
|  | | China | | | |  |  | |  | |  | |  | | | | 15.66 | | | 11.4 | | 12.99 | 13.33 |  |  |
| Clinical characteristics | | B+ ^a^ | | | | 205 | 260 | | 271 | | 276 | |  | | | | 67 | | | 59 | | 64 | 41 |  |  |
|  |  | B- ^b^ | | | | 234 | 290 | | 349 | | 325 | |  | | | | 40 | | | 31 | | 29 | 39 |  |  |
|  |  | SS+ ^c^ | | | | 77 | 81 | | 75 | | 85 | |  | | | | 31 | | | 32 | | 31 | 23 |  |  |
|  | | MDR-TB | | | | 1 | 6 | | 2 | | 3 | |  | | | | 0 | | | 0 | | 0 | 0 |  |  |
| TB treatment | | DOST | | | | 297 | 398 | | 457 | | 484 | |  | | | | 101 | | | 92 | | 92 | 80 |  |  |
|  |  | Died | | | | 1 | 2 | | 2 | | 3 | |  | | | | 0 | | | 1 | | 1 | 0 |  |  |
|  | | Transferred out | | | | 426 | 526 | | 457 | | 380 | |  | | | | 9 | | | 2 | | 3 | 2 |  |  |
|  | | Transferred-out rate | | | | 0.903 | 0.905 | | 0.707 | | 0.609 | |  | | | | 0.083 | | | 0.022 | | 0.032 | 0.024 |  |  |
|  | Lost to follow up | | |  | 8 | | | 9 | | 11 | | 15 | |  | | | 0 | | | 1 | 1 | | 1 |  |  |
|  | | Staying in Taiwan (ST) ^d^ | | | | 46 | 55 | | 189 | | 244 | |  | | 100 | | | | | 90 | | 92 | 82 |  |  |
|  | | Treat. completion (TC) ^e^ | | | | 46 | 48 | | 178 | | 233 | |  | | | 99 | | | | 89 | | 90 | 80 |  |  |
|  | | TC = 6-9 months ^e^ | | | | 42 | 48 | | 174 | | 232 | |  | | 99 | | | | | 89 | | 88 | 79 |  |  |
|  | | TC > 6-9 months ^e^ | | | | 0 | 0 | | 4 | | 1 | |  | | 0 | | | | | 0 | | 2 | 1 |  |  |
|  | | STT- completion rate ^f^ | | | | 0.913 | 0.873 | | 0.92 | | 0.95 | |  | | 0.96 | | | | | 0.989 | | 0.96 | 0.96 |  | |

1. B+: bacterial positivity, with positivity among 3 sputum smears or sputum cultures
2. B-: bacterial negativity, with no positivity among 3 sputum smears or sputum cultures
3. SS+: positivity among 3 sputum smears
4. Staying in Taiwan cases = all TB cases – transferred-out cases, including compulsorily repatriated cases
5. Treatment completion using 6-9-month regimens, i.e. a standard regimen or longer regimen
6. The treatment completion rate of cases staying in Taiwan = cases staying in Taiwan with treatment completion by a standard or longer therapy regimen / cases staying in Taiwan
